# Respiratory Failure Associated with Ascariasis in a Patient with Immunodeficiency

**DOI:** 10.1155/2016/4070561

**Published:** 2016-05-22

**Authors:** Lanocha Aleksandra, Zdziarska Barbara, Lanocha-Arendarczyk Natalia, Kosik-Bogacka Danuta, Guzicka-Kazimierczak Renata, Marzec-Lewenstein Ewa

**Affiliations:** ^1^Department of Haematology and Transplantology, Pomeranian Medical University, Unii Lubelskiej 1, 71-252 Szczecin, Poland; ^2^Department of Biology and Medical Parasitology, Pomeranian Medical University, Powstańców Wielkopolskich 72, 70-111 Szczecin, Poland; ^3^Department of Anesthesiology and Intensive Care, Pomeranian Medical University, Unii Lubelskiej 1, 71-252 Szczecin, Poland

## Abstract

In industrialized countries, risk groups for parasitic diseases include travelers, recent immigrants, and patients with immunodeficiency following chemotherapy and radiotherapy and AIDS. A 66-year-old Polish male was admitted in December 2012 to the Department of Haematology in a fairly good general condition. On the basis of cytological, cytochemical, immunophenotypic, and cytogenetic analysis of bone marrow, the patient was diagnosed with acute myeloblastic leukemia. On the 7th day of hospitalization in the Department of Haematology, patient was moved to the Intensive Care Unit (ICU) due to acute respiratory and circulatory failure. In March 2013, 3 months after the onset of respiratory failures, a mature form of* Ascaris* spp. appeared in the patient's mouth. This report highlights the importance of considering an* Ascaris* infection in patients with low immunity presenting no eosinophilia but pulmonary failure in the central countries of Europe.

## 1. Introduction

*Ascaris lumbricoides* (Linnaeus, 1758) is the most common soil-transmitted intestinal nematode affecting humans and causing significant medical problems, especially in developing countries. Globally, estimated 891.6 million people were infected with* A*.* lumbricoides* in 2010 [1]. In Europe, ascariasis is a rare condition affecting mostly rural citizens (1.2%) and people who are in regular contact with pigs for professional reasons. In Poland, the prevalence of* A. lumbricoides* in humans does not exceed 1% and concerns mainly children from rural areas [2]. Ascariasis generally occurs through hand-to-mouth ingestion of agricultural products or food contaminated with parasite eggs. Poor sanitation and inadequate sewage disposal play a key role in the maintenance and propagation of ascariasis [3]. Although not common in developed countries, ascariasis invasion is increasingly likely to be encountered by clinicians because of the growing rates of travel to developing countries and increased migration. Moreover, in industrialized countries, risk groups for parasitic diseases (e.g.,* A. lumbricoides* infection) include patients with immunodeficiency following chemotherapy and radiotherapy and AIDS [4, 5].

The pig roundworm* Ascaris suum* (Goeze 1758) has also been reported to be able to infect humans and develop into the adult stage. Both the morphology and development cycles of* A. lumbricoides* and* A. suum* are very similar and the identification of these species in the environment is very difficult [6]. Most infections with* A. lumbricoides* are asymptomatic. Clinical manifestations are different at each stage of the infection. This helminth usually lives in the small intestine but can also cause intestinal obstruction or perforation peritonitis, common in childhood [7]. Furthermore,* A. lumbricoides* can also migrate through ampulla of Vater to produce cholangitis, pancreatitis, cholecystitis, and hepatic abscesses in rare cases [8]. Second-stage larvae (L_2_) hatching from the invasive egg in the body pass through the intestinal wall and migrate via the portal vein system to the liver and then proceed to the lungs, where they may produce pneumonia. Symptoms include wheezing, dyspnea, nonproductive coughing, hemoptysis, and fever. The respiratory distress experienced during pulmonary ascariasis, referred to as Löffler's syndrome, occurs 4 to 16 days after ingesting embryonated roundworm eggs [9]. Skin urticarial rash may also accompany these symptoms. Chest X-rays reveal rounded infiltrates with peripheral eosinophilia [10]. Occasionally,* A. lumbricoides* may pass from the nose or the mouth during vomiting [11]. In this study, we present an unusual ascariasis and respiratory failure in a patient with acute myeloblastic leukemia (AML).

## 2. Case Presentation

A 66-year-old male was admitted in December 2012 to the Department of Haematology in a fairly good general condition. In an interview during admission, the patient reported coughing, a recurrent low-grade fever, night sweats, malaise, and weakness. Physical examination revealed bilateral lymphadenopathy: cervical (up to 2 cm), axillary to 1.5 cm, numerous inguinal nodes (up to 2–2.5 cm), single crackles at the base of both lungs, no hepatomegaly, and mild splenomegaly 1 cm below costal margin. Laboratory studies showed increased inflammatory parameters, serum lactate dehydrogenase (LDH), anemia and thrombocytopenia, blasts in the blood smear, and negative bacteriological results concerning the presence of aerobic and anaerobic bacteria. Microscopic examination of stool was negative for helminthic ova, and examination of feces was recommended to be conducted several times. No helminth eggs were found. Chest X-ray revealed no indication of infiltrates in the lungs or pneumothorax. On abdominal ultrasonography, the liver was not palpable, while the spleen was uniform but enlarged; in the long axis, it measured 133 mm. On the basis of cytological, cytochemical, immunophenotypic, and cytogenetic analysis of bone marrow in December 2012, the patient was diagnosed with acute myeloblastic leukemia. On the 7th day of hospitalization in the Department of Haematology, patient was moved to the Intensive Care Unit (ICU) due to acute respiratory and circulatory failure. The patient displayed violent dyspnea and a cherry-colored scalp and neck, along with high anxiety and respiratory effort, tachypnea 43/min, and auscultation of the lungs: individual crackles at the base of the lungs, including single crackles towards the angles of the blades and increased vesicular murmur over the whole lung. HR is 130/min, RR is 150/110 mmHg, and oxygen saturation is 92–94% on an oxygen mask with a reservoir, at a flow rate of 15 L/min. Before admission to the ICU, morphine, steroids, and diuretics were administered. This resulted in little improvement and tachypnea 20–25/min. Blood gases did not correlate with the clinical condition of the patient, the clinical picture suggestive of pulmonary embolism. In the following minutes, an increased severity of changes was noticed during the auscultation of the lungs; shortness of breath remained with an extended expiratory phase. The patient was intubated and treated in the ICU. After a slight improvement in general condition, the patient was transferred to the Department of Haematology but was still in a general poor condition. In a short time, the patient was returned to the ICU. A build-up of heart failure was observed and clinical symptoms of pulmonary edema were observed; these were treated in a conventional manner and a transient improvement was obtained. However, in a short time, once again respiratory problems returned, with a decrease to 76% oxygen saturation despite constant oxygen flow to the oxygen mask with a reservoir (15 L/min), and the occurrence of numerous changes was recorded during the auscultation of the lungs. Respiratory failures were accompanied by losses of consciousness. Chest X-rays showed bilateral multiple scattered airless changes, most likely associated with inflammations.

After improvement in general condition in January 2013, the patient was transferred to the Department of Haematology to measure bone marrow cytology: 83.6% myeloblasts. The patient was qualified for palliative therapy with hydroxyurea because of poor general condition and respiratory and heart failure. Two months after the onset of pulmonary failures, chest X-ray inspections showed no infiltrates in the lungs, wherein in the bottom of the left lung and the tops of both the lungs discreet cicatricial changes, and echocardiography EF (ejection fraction) was 55% (previously 35%), nonenlarged heart. The patient was qualified for intensive chemotherapy: remission induction therapy DA (daunorubicin with cytarabine) 2nd group stratification was at >60 years of age according to the PALG protocol. Chemotherapy was complicated by sepsis, probably catheter-related, with an etiology of* Staphylococcus* epidermidis.

**Figure 1:**
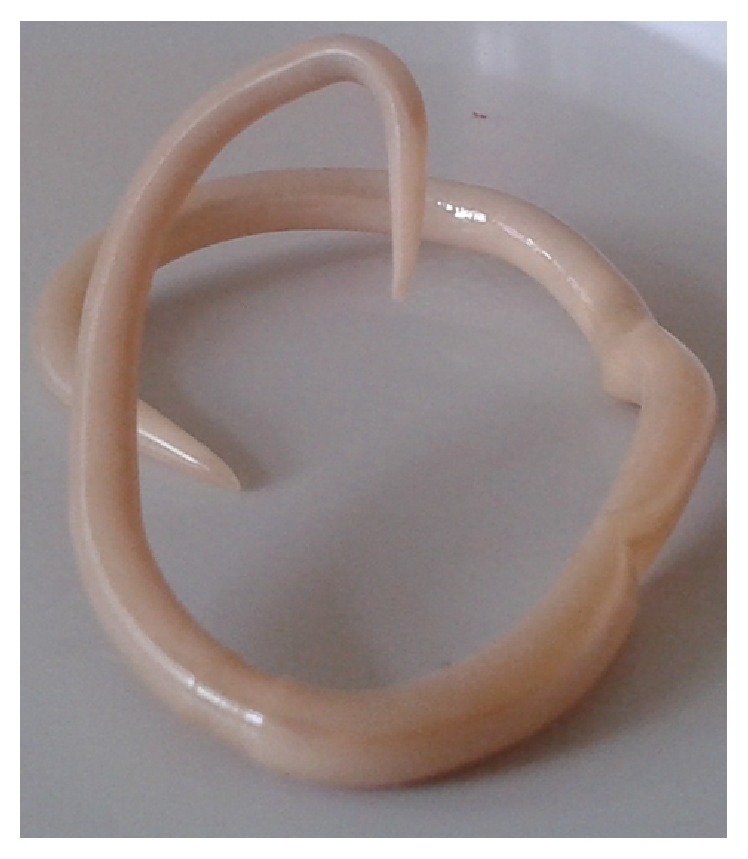
Adult male of* Ascaris lumbricoides*.

In March 2013, 3 months after the onset of respiratory failures, a mature form of* Ascaris* spp. appeared in the patient's mouth (Figure 1). Stereomacroscopic examination showed that it was a male roundworm, 15 cm long and 2.5 mm diameter. Pyrantel was administered orally. The patient responded well to the antiparasitic treatment and showed no gastrointestinal symptoms. In an interview with the patient, he denied eating unwashed fruits and vegetables, but two weeks prior to the onset of respiratory failure (December 2012) he had removed the feces of pigs at a farm.

## 3. Discussion

This report highlights the importance of considering an* Ascaris* infection in patients with low immunity presenting no eosinophilia but pulmonary failure in the central countries of Europe. Populations at risk for* A. lumbricoides* and* A. suum* infestation are those who have stayed in areas with suboptimal sanitation, practiced poor personal hygiene, and have a poor educational background, especially in developing countries. Ascariasis in humans can cause gastrointestinal and respiratory symptoms and severe complications requiring surgical intervention [12]. In the human host, the worm can establish residence in the gastrointestinal region from the stomach to the ileocecal valve, but up to 99% of the cases reported have worms localized to the jejunum and proximal ileum [13, 14]. Furthermore there are some reports of erythema nodosum and severe sepsis after a spontaneous abortion in a patient with coexisting ascariasis [15, 16].

Clinical manifestations are different at each stage of the infection. Parasites migrate from the small intestine to the pulmonary circulation where they mature and destroy capillaries and alveolar walls [17]. Larval pulmonary migration is generally asymptomatic. However, symptomatic pulmonary ascariasis is characterized by a dry cough, low fever, dyspnea, bronchial asthma, and wheezing. The respiratory distress experienced during pulmonary ascariasis is referred to as Löffler's syndrome and occurs 4 to 16 days after ingesting embryonated roundworm eggs [18].

In many cases, pulmonary ascariasis in chest X-rays shows fleeting infiltrates that may develop peripheral confluence and intra-alveolar hemorrhage, and exudate may also be present (associated with larvae migration) [9, 19, 20].

In our patient, arterial-blood gas analysis did not correlate with the clinical condition of the patient and the clinical picture suggested pulmonary embolism. Two months after the pulmonary failure, a chest X-ray showed no infiltrates in the lungs and pneumothorax. However, discreet cicatricial changes were observed in the lower left lung and the tops of both lungs. It cannot be excluded that the presented case of respiratory failure in a patient with AML was probably provoked by the infestation with roundworm.

Two cases of pulmonary ascariasis in Austrian males in 2010 have been reported. Both patients demonstrated dyspnea, a nonproductive cough, fever, and eosinophilia (19 and 26%). One of the patients was in remission of b-cell non-Hodgkin lymphoma. Serology for* Ascaris* was positive twice in both patients, while microscopic examination of stool was negative for helminthic ova [9].* Ascaris* worms may enter nasogastric and endotracheal tubes and block their lumen [13]. There are case reports in the literature of fatal outcomes from blockages of endotracheal tubes by roundworms [21, 22].

Two to three months are required between ingestion of the infective eggs and moulting to the adult male or female. Females may produce 250,000 eggs per day which are excreted in the feces of the host.* A. lumbricoides* and* A. suum* are geohelminths (transmitted soil nematodes); eggs become invasive after 3-week residence in the soil under suitable environmental conditions [23]. They are resistant to environmental factors thanks to their chitinous sheath. Unembryonated ova enter the environment via feces and can remain viable in the soil for up to 15 years [18]. As in our patient, in many cases usually there is a lack of eggs in stool samples in both roundworm species until 2 to 3 months after the pulmonary symptoms occur, unless the patient was already harboring a patent infection. When infections occur with only male worms, no eggs are formed. Diagnosis is most commonly made by finding eggs in the stool; occasionally an adult worm is passed in the stool. Adults of* Ascaris* spp. worms expelled from the anus or the mouth could be identified using the classical characteristics of the worm. During the early transpulmonary migratory phase, larvae can be found in sputum or gastric aspirates [18]. In this case, this diagnosis was considered when an adult male worm was found in the patients mouth. Esophagus ascariasis is extremely rare because the esophagus is not a normal habitat of* Ascaris *spp., as it prefers an alkaline environment and rarely travels from the jejunum and duodenum to the stomach (an acid environment) and then to esophagus [24].

Patients with some type of immunocompromised condition and those submitted to immunosuppressive therapy have an increased probability of acquiring parasitic infections, generally with a high degree of severity [25]. The varied clinical picture of the parasitic infection in people with low immunity results in a number of cases that are not recognized. Parasitic infestations in states of immune deficiency may pose a serious threat to health and even life, with sudden scattered symptoms affecting many organs. Nonopportunistic intestinal parasites such as hookworms,* Opisthorchis viverrini*, and* A. lumbricoides* were common in HIV-infected patients after chemotherapy regardless of immune status [26]. This case study is nontypical, because with no increase in eosinophil count we did not suspect ascariasis. Eosinophilia often occurs in verminuous diseases, in particular tissue in which such parasites are readily available to the eosinophils migrating within tissues. In the early stages of invasion of the respiratory tract, high eosinophilia is common (1.0–3.0 · 10^9^/L), and the larvae of* Ascaris* spp. occur occasionally in the sputum. High eosinophilia is associated mainly with the wandering larvae. Over time it falls below 1.0 · 10^9^/L and is not a pathognomonic symptom in ascariasis induced by mature parasites [27]. Peripheral blood eosinophilia was absent in this study on admission and the diagnosis was partly misled by immunodeficiency in the patient because of acute myeloblastic leukemia.

*Ascaris lumbricoides* and* A. suum* in humans are transmitted through ingestion of agricultural products or food contaminated with parasite eggs. In Poland, human infection with* A. suum* and pig infection with* A. lumbricoides* are possible, and both these species are considered together in the epidemiological evaluation of the environment [28]. The prevalence of* A. suum* in pigs has declined over the last decades as well; infections in humans may now be more common in the western world as contact with pig feces (as manure spread) may occur frequently [9].

*Ascaris lumbricoides* and* A. suum* may constitute two different but closely related species or may represent host-associated subpopulations or races of the same species [29, 30]. Leles et al. [6] suggested that the origin of* Ascaris* spp. is still not well understood. Studies obtained from experimental infections and molecular methods have been inconclusive, and to 2016 none of the published studies answered the question of whether* A. lumbricoides* and* A. suum* are truly distinct species. Researchers including Alves et al. [31] and Betson and Stothard [32] discussed that the use of mitochondrial markers or DNA barcoding approaches to infer species relationships and transmission dynamics for* Ascaris* is controversial. There are in the literature publications about pig-associated haplotypes among* Ascaris* worms collected from humans who live in areas without pigs, suggesting retention of ancestral haplotypes. In contrast based on microsatellites, these parasites looked like human-associated* Ascaris* [33].

## 4. Conclusion

This case of respiratory failure in a patient with AML, probably induced by human* Ascaris* infestation, is rather rarely discussed. It should be noted that an exact diagnosis of the patient and a search for nonspecific common etiological factors (including ascariasis) is required to achieve good outcomes and improve the patient's condition, while not excluding cases of patients with low immunity. In conclusion, physicians should pay more attention to the possibility of this disease and reduce the risk of misdiagnosis in this regard.

## Abbreviations

AML: Acute myeloblastic leukemia
DA: Daunorubicin with cytarabine
EF: Ejection fraction
HR: Heart rate
ICU: Intensive Care Unit
LDH: Lactate dehydrogenase
L_2_: Second-stage larvae
PALG: Polish Adult Leukemia Group
RR: Respiration rate.
